# Breast Silicone Gel Implants versus Autologous Fat Grafting: Biomaterials and Bioactive Materials in Comparison

**DOI:** 10.3390/jcm10153310

**Published:** 2021-07-27

**Authors:** Pietro Gentile

**Affiliations:** 1 Department of Surgical Science, “Tor Vergata” University, 00133 Rome, Italy; pietrogentile2004@libero.it; Tel.: +39-3388-5154-79; 2 Academy of International Regenerative Medicine & Surgery Societies (AIRMESS), 1201 Geneva, Switzerland

**Keywords:** breast augmentation, breast fat grafting, breast implants, breast remodeling, plastic surgery

## Abstract

In the last 20 years, surgical procedures in breast remodeling during mammoplasty have been deeply modified with a gradual shifting from an invasive intervention using definitive implants (DIs) to a more conservative autologous fat grafting (AFG). AFG has been used for many years as bioactive material through the Lipofilling technique and as a bioactive scaffold when it was enriched with adipose-derived stem cells (ASCs), while DIs have been considered physiologically inert biomaterials with low toxicity. The paper aimed to compare the breast remodeling results obtained in the DI group (55 patients) for hypoplasia correction with those of the ASC-enhanced AFG group (50 patients), also analyzing the influence of breast and chest deformities (tuberous breast, volume, and nipple–areola complex asymmetry, pectus excavatum and carinatum) in the cosmetic outcome. A retrospective, case-control study was conducted. The pre-operative analysis was performed with an accurate clinical evaluation, a photographic assessment, and an instrumental evaluation based on magnetic resonance imaging, mammography, and ultrasound. Of patients treated with DIs 89% (n = 49) showed excellent cosmetic results after 1 year compared with the patients treated with AFG, who showed the same results in 64% (n = 32) of cases. The naturalness of the results in the AFG group was higher than that in the DI group (*p* < 0.0001 vs. DI group). DIs and AFG were safe and effective in this case series treated. The AFG group showed more natural results, allowing the treatment of patients with pectus excavatum, while DIs showed the more evident and lasting result.

## 1. Introduction

Breast augmentation is a delicate procedure, and it relies on a deep knowledge of the relationship between the different anatomical structures of the chest as muscle, gland, fat, soft tissues, derma, and skin. This concept is truer when the mammoplasty is performed in underweight patients since it is necessary to perform a submuscular implant positioning. The breast tissues’ thickness plays a pivotal role in determining the treatment choice of the anatomic plane in which the implant will be positioned (sub-glandular, sub-muscular, or dual plane). As a result, it is necessary to know how to intervene to obtain the desired outcomes, via the definitive implants (DIs) use [1].

In the last 20 years, surgical procedures in breast augmentation have been deeply modified with a gradual shifting from exclusive use of biomaterials represented by DIs to a less invasive mammoplasty without DIs based exclusively on autologous fat grafting (AFG) [2]. This latter interesting strategy of breast remodeling and augmentation is based on minimal manipulation of fat tissue via centrifugation, filtration, or via enzymatic digestion using human collagenases [2].

The aim is to obtain a purified fat tissue enriched with adipose-derived stem cells (ASCs), improving fat graft maintenance. ASCs are in subcutaneous adipose tissue Stromal Vascular Fraction (SVF), which contains a heterogeneous mesenchymal cell set [3]. SVF may be extracted from adipose tissue using both an enzymatic digestion, to remove most of the hematopoietic cell population from the SVF cells (SVFs), or mechanical digestion based on a combined procedure of centrifugation and filtration [4].

Therefore AFG, a biological autologous tissue containing several important cellular components (adipocytes and mesenchymal stem cells), extracellular matrix (ECM), vessels, and nerves [3], can be considered either as “bioactive material” when it is grafted into the soft tissue defects aiming to improve the volume and tissue quality or as “bioactive scaffold” when it is enriched with ASCs. In this last case, AFG acts also as a scaffold for the ASCs, representing an autologous biological matrix (cellular and extracellular) in which these cells may be incorporated and transported to improve the fat graft maintenance through an autologous regenerative approach prevalently based on adipogenesis and vascularization improvement.

The main limits of AFG are the resorption and the controversial breast cancer relationship in obese patients. The most important and recent studies on breast augmentation procedures performed with AFG described a 58% maintenance of the fat volume after 3 years when AFG was enriched with ASCs, compared to AFG without ASC addition, who showed 29% maintenance [2].

The adopted techniques (AFG enhanced with ASCs and/or not enhanced) did not represent a significant risk factor for tumor recurrences as confirmed in recent clinical trials [3]. As is known, AFG releases adipokines, which are modulated during obesity, and they could have “remote” effects on breast carcinogenesis [3]. A growing body of evidence indicates that only obesity has a main oncological risk factor and peri-tumor fat tissue as well as its progenitor cells are a source of pro-tumor factors [3]. In this field, Gentile et al. [3] reported the percentage of recurrences in three different groups of patients in which the study group (SG) was treated with AFG enhanced with ASCs for breast reconstruction, control group 1 (CG1) was treated with non-enriched AFG, and control group 2 (CG2) was not treated. In a group of seven patients (CG2) (all affected by obesity), three recurrences (two systemic and one local) were recorded, as compared with four recurrences (three systemic and one local) in an SG including 121 patients and five recurrences (two systemic and three local), while CG1 was composed of 50 patients [3].

On the other hand, the major side effects related to the use of DIs, such as implant displacement, deformities, reject, wrinkling, rippling, and the most recent association with anaplastic lymphoma, led several plastic surgeons to develop new and non-invasive strategies for breast remodeling [1].

DI biomaterials are frequently used in breast remodeling, thanks to their physiological inertness and low toxicity. However, capsular contracture remains a concern in long-term follow-up. Additionally, an inappropriate resection of the pectoralis major muscle can lead to severe problems, such as the “jumping breast” phenomenon [1,5]. The accurate evaluation of the breast soft tissue, prevalently represented by muscle, gland, fat, and patients’ expectations, plays a pivotal role in the treatment choice (DIs or AFG) [1,2,4,5]. Moreover, it becomes necessary to evaluate the fat amount in the flanks, abdomen, and thighs when the plastic surgeon is selecting AFG. Underweight patients are not considered suitable for this kind of procedure.

For the abovementioned reasons, this study aims to compare the cosmetic results obtained in breast hypoplasia correction using DIs with those obtained using AFG, analyzing the influence of breast and chest deformities (tuberous breast, volume, and nipple–areola complex (NAC) asymmetry, pectus excavatum and carinatum) and the low body mass index (BMI), in the cosmetic outcome. The related advantages and disadvantages of each strategy are reported.

## 2. Methods

### 2.1. Study Overview and Data Definitions

A retrospective case-control study, classified as evidence-based medicine (EBM) level 3, was performed fully respecting the Declaration of Helsinki and internationally consented ethics in clinical research [6]. The Strengthening the Reporting of Observational Studies in Epidemiology (STROBE) checklist [7] was applied to develop a quality assessment. The patients involved in the study compiled and signed a detailed informed consent including all risks, benefits, and alternative procedures, before any treatment. The protocols adopted were performed following the European rules (1394/2007 EC) and EMA/CAT recommendations (20 June 2014 EMA/CAT/600280/2010 Rev 1). This paper was the result of a research contract between the author and the “Tor Vergata” University, approved by Rectoral Decree R.D n. #1467/2017, continued in associate professor contract #13489/2021.

### 2.2. Patient Population

**Figure 1:**
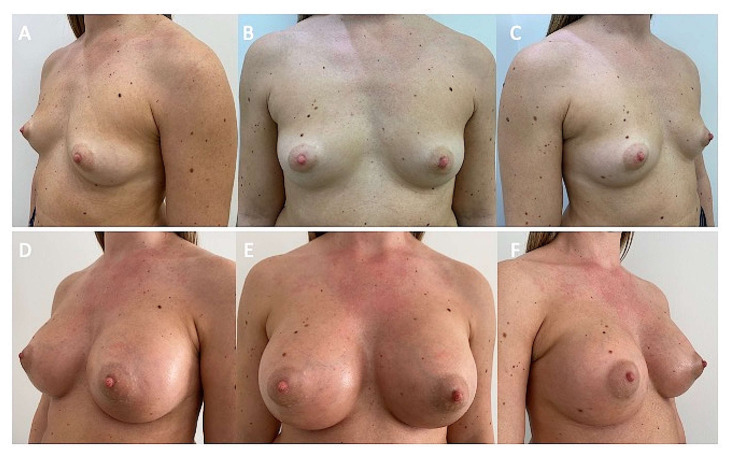
A 30 year-old-female, affected by a moderate grade of bilateral breast hypoplasia, was treated with definitive implants. (**A**) Pre-operative projection in ¾ left view. (**B**) Pre-operative projection in frontal view. (**C**) Pre-operative projection in ¾ right view. (**D**) Post-operative projection after 1 year (T6) in ¾ left view. Definitive implants of 320 cc, round and smooth, were applied bilaterally under the glands—not sub-muscular/dual-plane. (**E**) Post-operative projection after 1 year in frontal view, evidencing the significant breast improvement after one surgical procedure. (**F**) Post-operative projection after 1 year in ¾ right view.

**Figure 2:**
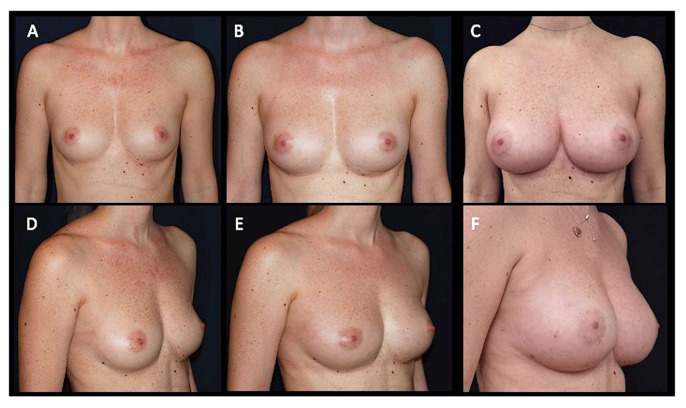
A 27 year-old-female, affected by a moderate grade of bilateral breast hypoplasia, was treated with autologous fat grafting. (**A**) Pre-operative projection in frontal view. (**B**) Post-operative projection in frontal view after six months (T5) and one procedure based on ASC-enriched AFG. An increase of 29.5 mm in the three-dimensional volume after 6 months (T5) was observed. (**C**) Post-operative projection in frontal view after 1 year (T6) and two procedures based on ASC-enriched AFG. An increase of 72.8 mm in the three-dimensional volume after 1 year (T6) was observed, evidencing a cosmetic result comparable with that obtained by the definitive implants. (**D**) Pre-operative projection in ¾ right view. (**E**) Post-operative projection in ¾ right view after six months (T5) and one procedure based on ASC-enriched AFG. (**F**) Post-operative projection in ¾ right view after 1 year (T6) and two procedures based on ASC-enriched AFG evidencing the naturalness of the outcome.

Over ten years (2008–2018), 55 patients affected by breast hypoplasia (n = 30 suffering a moderate grade (Figure 1A–C) of bilateral hypoplasia (BH), n = 10 with a high grade of BH, n = 12 with a low grade of BH, and n = 3 with unilateral breast hypoplasia (UH)), were treated with DIs for breast aesthetic augmentation. The DI group comprised 55 females aged 18–61 years (average age 39.5). Pre-menopausal patients were 39 (71%). A long-term follow-up of cosmetic results was analyzed comparing the DI group’s outcomes with those shown by a group of 50 patients treated with AFG. The AFG group comprised 50 females aged 18–58 years (average age 38), all affected by breast hypoplasia (n = 7 affected by a high grade of BH, n = 28 with BH of a moderate grade (Figure 2A,D), n = 10 with BH of a low grade, and 5 patients affected by UH). Pre-menopausal patients were 38 (76%) and 39 (71%), respectively, in the AFG group and DI group. An accurate pre-operative screening based on a full clinical evaluation, a photographic and instrumental assessment performed by magnetic resonance imaging (MRI), ultrasound (US), and mammography (MG) was performed in all patients enrolled. The patients were strictly instructed about the full regimen of post-surgery recommendations (limit arm movements, do not strain, sleep in a semi-sitting position, and do not remove the applied elastic-compressive sheath, during the following 20 days in both DI and AFG groups) as important factors to obtain optimal outcomes in breast augmentation surgery, independently by the surgical strategies.

### 2.3. Inclusion and Exclusion Criteria

The following inclusion criteria were considered: age 18–70 years old, history of high, moderate, and low grades of BH and UH, a BMI between 18 and 35 kg/m^2^. Additional inclusion criteria in the AFG group were sufficient fat in the abdomen, thighs, flanks, and inner knee regions (sites of fat harvest). The following local and systemic exclusion criteria were considered: systemic exclusion criteria (bone marrow aplasia, anti-aggregating therapy, sepsis, cancer, and uncompensated diabetes), local exclusion criteria (reconstruction with expanders, reconstruction with first stage AFG followed by DIs, reconstruction with implants followed by an AFG session, previous breast surgeries, cancer loss of substance and uncontrolled comorbidities). Smoking or genetic disorders were not considered as exclusion criteria.

### 2.4. Clinical Data Assessment and Quality Checks

The following parameters were recorded in the database: age, demographic data, BMI, surgical treatment performed, side effects (Table 1). Every patient was evaluated by a multidisciplinary team including a plastic surgeon, a radiologist, and a psychologist.

Every patient underwent three clinical evaluations performed by the plastic surgeon (P.G.). During the first visit (lasting not less than 60 min), every patient was clinically evaluated by performing a strict anamnesis and posing several questions regarding her expectations. Clinical grade (high, moderate, and low) of BH/UH was evaluated. The distribution of body fats was analyzed. The surgical procedure to adopt was established considering the patient’s expectation, clinical evaluation, and inclusion and exclusion criteria. Risks and side effects related to the surgical strategy identified were accurately discussed. Blood and instrumental tests were contextually required. The second clinical evaluation aimed to evaluate the patient’s suitability, based on blood and instrumental analysis results. In particular, the radiologist evaluated the instrumental analysis outcomes, the psychologist the eventual presence of body image perception disorder, dysmorphophobia, personality, and/or mood disorders. The third clinical meeting was used to sign the informed consent (P.G. and patient) and to clarify any doubts.

During the first 3 years, patients were analyzed at 1, 3, 7, 12, 24, and 48 weeks, and then annually for 3 years by clinical examination at 3 weeks (T2), 6 months (T5), 12 months (T6), and then annually until the third year after the last procedure by MRI and US. Abnormal clinical findings were further investigated. Deformities and asymmetries were documented by clinical examination and instrumental tests. In detail, a clinical evaluation (measurement of breast volume), pictures (hypoplasia and deformities evaluation), and an instrumental evaluation (MRI scans and the US and MG) were performed to estimate the degree of breast hypoplasia.

Additionally, quality checks were performed in all patients based on the following criteria:

− Surveys related to the patient’s grade of satisfaction with resulting texture, softness, contours and augmentation volume; availability to undergo the procedure again; willingness to recommend the treatment to friends; satisfaction with sufficient information about risks and complications of treatments (range 1 to 6: excellent [1]; very good [2]; good [3]; sufficient [4]; poor [5]; very poor [6]) (Appendix A);
− Clinical evaluation based on Physician Global Assessment score (excellent, good, discreet, enough, poor, inadequate);
− Clinical evaluation based on Patient Global Assessment score (excellent, good, discreet, enough, poor, inadequate);
− Visual Analog Scale (VAS) (range 1 to 6: excellent [1]; very good [2]; good [3]; sufficient [4]; poor [5]; very poor [6]);
− Additional factors/variables, such as volume asymmetries, breast and chest deformities, NAC asymmetries, pseudoptosis, pectus excavatum, and carinatum, low BMI, (presence: high, moderate, and low degree, or absence);
− Adverse effects signaling (presence or absence).

### 2.5. Breast Augmentation with Definitive Implants: Prosthesis and Surgical Technique

Definitive biomaterial breast implants are classified in two different categories, saline-filled or silicone-filled biomaterials, but both present an outer silicone shell that can be textured or smooth. The Food and Drug Administration (FDA) approved the use of silicone gel implants to increase breast size in any patient over the age of 22, and, if it appears widespread, their “off-label” use in younger females (under 22) when indicated for the patient. Silicone gel implants present a softer touch and/or more natural feel, and they show less rippling of the edges, finding a greater indication in thin patients with small amounts of soft tissue coverage compared with saline implants. For the abovementioned reasons, the author used in all cases silicone gel implants. The DIs may be placed in two different anatomic planes: sub-glandular and sub-muscular, in which the implants are inserted either above or below the pectoralis muscle, respectively, with related advantages and disadvantages to each approach. In the DI group, the prostheses were positioned sub-glandular in 71% (39 patients) and sub-muscular in 29% (16 patients).

The patient’s anatomy—in particular, chest and breast shape, breast volume, and breast tissue thickness—the patient’s need as well as surgeon preference play a pivotal role in the implant positioning planning (sub-muscular/sub-glandular/dual plane).

Three surgical incisions were described to access the identified plane: infra-mammary, trans-axillary, and peri-areolar. The incisions adopted in the DI group were inframammary (93%—51 patients) and peri-areolar (7%— 4 patients).

General anesthesia is most common for this procedure, but it can also be done under sedation. All DI patients (100%) were treated with general anesthesia.

Breast augmentation with DIs generally takes 60 to 120 min to perform. Recovery is usually rapid with a return to light work and activity within two weeks. Returning to full activity may take up to six weeks.

### 2.6. Breast Augmentation with Definitive Implants: Surgical Planning and Complicating Factors

All DI group patients received round DIs, except for three who received anatomical DIs at their request. The profile (moderate or high) and surface (smooth or textured) of the DIs were decided according to pre-operative breast volume (high, moderate, or low degree of breast hypoplasia), breast tissue thickness and coverage, and patient expectations. If the patient had nipple asymmetry (in the case of vertical or lateral NAC malposition) or had tuberous breasts with hypoplasia, a peri-areolar incision was performed. In all other patients, an inframammary incision was chosen. Initially, the author’s preference was for the smooth implants for dual-plane pockets and the textured implants for sub-glandular pockets. Currently, smooth DIs are used for both. The size of the DIs used was between 150 and 320 ccs. The choice of the DIs (both anatomical and round) and the related positioning plane was made based on the availability of the tissue, with “custom made” methods. Drains were applied in every case. If needed, vertical and radial cutting scoring of the inferior gland was performed to expand the breast inferior segment or in cases of tuberous breast.

Standard front left/right lateral and left/right oblique pictures were collected preoperatively and postoperatively. Any evident abnormality in breast volume (high grade of hypoplasia or unilateral breast hypoplasia), NAC position, breast shape (tuberous), or the presence of pseudoptosis, underweight patients, pectus excavatum, and carinatum were defined as a non-optimal result, here identified and called “suboptimal result.” The deformities correlation coefficient (DCC) was also calculated to measure their influence. The patients’ clinical features and deformities were deeply analyzed, aiming to identify a list of factors that could have downgraded the outcome of the procedure, identifying the incidence of each of these deformities. The number of pre-operative deformities for each patient was determined (Table 2), and the relationships between the non-optimal surgical (sub-optimal) results and these deformities were evaluated. Pre-operative deformities were considered complicating factors and then classified into major or minor factors. Major complicating factors were those that singularly led to a suboptimal outcome, and minor factors were those that led to a suboptimal outcome if they were present in combination (Table 3).

### 2.7. Breast Augmentation with Autologous Fat Grafting: Surgical Injection

**Figure 3:**
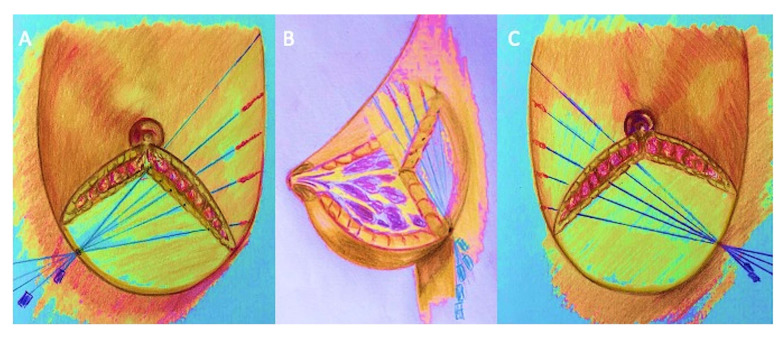
Painting of the “Gentle technique” for autologous fat injection. (**A**) Access point in lower-external quadrant located at 247.5° of the breast. (**B**) 3D painting of fat injection into the breast through three inframammary fold access located at 135°, 180°, 225°. The autologous fat injection was always performed in the infra and sub-glandular plane—not in the muscle. (**C**) Access point in lower-internal quadrant located at 112.5°of the breast.

To improve breast volume the AFG was injected into 7 different anatomic regions: upper and lower lateral and internal quadrant, peri-areolar region, lower and upper pole. In all AFG patients the anatomic region destined to receive the AFG was decided based on the necessary corrections analyzed through clinical assessment and MRI scans. The AFG injection was performed using the “Gentle technique” [8] (Figure 3A–C) based on a slow and gentle injection implanting linear deposits of AFG in the supra-fascial, retro-glandular and intra-glandular space using a 1.5 mm cannula [8,9]. In all AFG group patients, AFG was enriched with ASCs and used both as “bioactive material” (aiming to improve soft tissue volume) and “bioactive scaffold” (aiming to incorporate and transport ASCs to improve the fat graft maintenance). For the abovementioned reasons, the ASC-enriched AFG was implanted as bioactive material scaffold in the subcutaneous space (not into the pectoralis major muscle and parenchyma) in multiple tunnels with slow and controlled movements through different entrances (infra-mammary folds (located at 135°, 180°, 225°), higher external quadrant (292.5°), lower-external quadrant (247.15°), higher internal quadrant (67.5°) and lower-internal quadrant (112.5°)) to underline the importance of a non-traumatic procedure to maximize the integrity of the grafted tissue and to maximize the contact surface between the AFG and the host’s capillaries [4,8]. Two 10 mL syringes for every hole (about 20 mL of AFG), for a total of about 140 mL of ASC-enriched AFG for the side, were injected. Contextually, the ASC-enriched AFG was also injected via peri-areolar entrances located at 45°–135° and 225°–315° on the areolar circumference using 1–2 mL syringes through three or four tunnels arranged radially around the skin access. The amount of ASC-enriched AFG inserted through these accesses was about 50–80 mL, according to the breast needs. The procedure was repeated on the other side, and at the end the patient was checked for any asymmetry and deformities that were meticulously corrected. According to the patient’s needs, 80–280 mL (average, 180 mL) of fat grafting was injected for each breast, for a total of 360 mL (range, 160–560 mL) per patient.

Breast augmentation with AFG is considered an operation that generally takes 120 to 180 min to perform. Recovery is very rapid with a return to light work and activity within a week. Returning to full activity may take up to three weeks.

### 2.8. Breast Augmentation with Autologous Fat Grafting: Fat Processing

The AFG enrichment with ASCs was based on two different “one-step” processes: ASC isolation and extraction from adipose tissue and AFG purification and enrichment.

The ASC isolation and extraction may be performed using enzymatic or mechanical digestion as introduced briefly. The enzymatic digestion was performed in 27 patients (49%) using Celution™ 800/CRS System (Cytori Therapeutics Inc., San Diego, CA, USA, http://www.cytoritx.com). In this case, a liposuction (675 mL average in all patients—range 250 mL/1100 mL) in the abdominal region and/or flank and thighs using 3 mm cannulas was performed. The first half of the adipose tissue collected (230 mL average) was aseptically placed into the tissue collection container where blood and free lipid were removed via a wash cycle. Then the Celase™ 835/CRS reagent was added to enzymatically digest the adipose tissue, permitting release of SVFs containing ASCs. After additional wash and centrifugation cycles, 5 mL of SVF suspension containing ASCs and SVFs was obtained and extracted from the system. The second remaining half of adipose tissue (445 mL average) was added to the collection container to undergo a washing step. Once completed, 360 mL of purified AFG was obtained. At this point, 5 mL of SVF suspension was added, obtaining a 365 mL average of ASC-enhanced fat tissue, ready for grafting, that the author called ASC-enriched AFG. The enzymatic digestion process and related AFG enrichment were completed within 160 min. The mechanical digestion was performed in 23 patients (46%) using Fatstkit (CORIOS Soc. Coop, San Giuliano Milanese, Italy). In this case, the first amount of adipose tissue (80 mL) was collected in a specific bag as a collection container. Then the fat was harvested in four 20 mL Luer-look syringes to undergo a centrifugation cycle at 1700 RPM per 10 min. In the end, 40 mL of suspension was further filtered through a 120 μm filter. A 20 mL of the SVF suspension containing ASCs and SVFs was obtained. The second amount of fat (480 mL average) was purified via a centrifugation cycle at 1200 RPM for 10 min. An average total of 340 mL of purified AFG was collected and added to 20 mL of SVF suspension previously obtained. At the end, 360 mL of ASC-enhanced AFG was obtained. The mechanical digestion process and related AFG enrichment were completed within 60 min. In every case, The ASC-enriched AFG was transferred into 10 mL Luer-Look syringes and aseptically re-injected into the patient using specific 1.5-mm-diameter micro-cannulas for implantation.

### 2.9. Evaluation of Fat Amount to Inject into Each Breast

**Figure 4:**
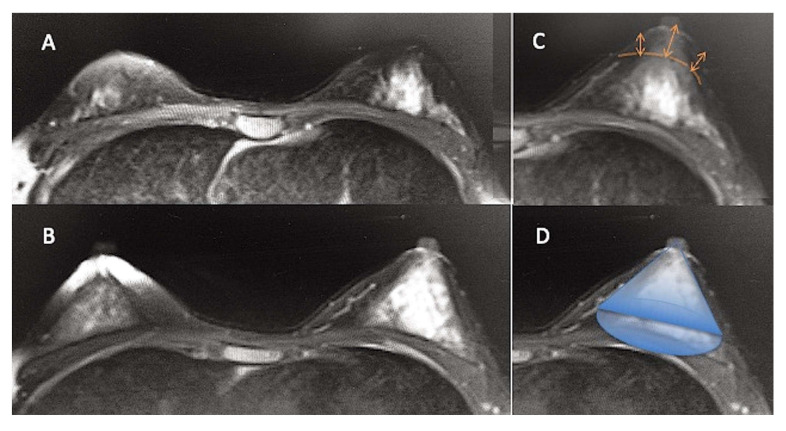
Magnetic resonance imaging (MRI) of a patient treated with AFG. (**A**) The pre-operative situation in a patient affected by bilateral breast hypoplasia. (**B**) The post-operative situation at T5 (6 months) after one AFG treatment with an evident improvement of bilateral breast volume. (**C**) Overlapping of pre-and post-op MRI images with the volume increase measurement detail of 29.5 mm (arrows). (**D**) Overlapping of a geometric cone on augmented breast to note the similar aspect of the breast to a cone.

A careful anamnesis (patient’s expectations), a clinical evaluation (measurement of breast volume), pictures (hypoplasia and deformities evaluation), and an instrumental evaluation (MRI scans) (Figure 4A) were performed to estimate the optimal volume of fat to graft. A 1.5-Tesla MRI (Hitachi, MS, Echelon Oval, Tokyo, Japan) was used to produce 3-mm-thick slice images, analyzed by OsiriX software, 32 bits, free version (Pixmeo, CA), to calculate breast volume. Two calculations were conducted per examination, and the determined average was taken as the final breast volume. Based on the acquired MRI scans, volumetric fat site assessments of the breasts were also calculated, utilizing as edges the anterior axillary line, anterior margin of the pectoral muscle, Medio-sternal line, skin, and nipple. They were analyzed on a separate workstation (ADW 4.0; GE Medical Systems, Milwaukee, WI, USA) employing a 3D reconstruction. Every breast was considered a geometric “cone” (Figure 4D), and, for this reason, the formula Volume = π × r^2^ × h / 3 (base area per height, divided by 3) to evaluate the initial breast volume and the optimal volume of fat to inject into it was applied [2], to convert the initial amount of fat in milliliters to a volume expressed in cm^3^ [2]. (For example, in a breast with a radius (r) of 6 cm and height (h) 5 cm, Cone Volume = 3.14 × 36 × 5= 565.2 / 3 = 188.4 cm^3^. Thus, the amount of fat graft injected will be 188.4 mL).

### 2.10. Clinical Evaluation Assessment

Clinical results obtained in DI and AFG patients were evaluated through a Physician Global Assessment score and Patient Global Assessment score. The Physician Global Assessment was based on clinical observation, using a six-value scale (excellent, good, discreet, enough, poor, inadequate). The Patient Global Assessment used the same six-value scale. The factors/variables considered were breast volume maintenance, naturalness, need to repeat the treatment, final breast volume, pain, side effects, major complicating effects.

### 2.11. Statistical Analysis

A comparison between DI and AFG groups was done with the Student’s *t*-test or Mann–Whitney for the self-assessment questionnaire. The data are expressed by mean (range), median (range), and percentages. A two-tailed *p*-value less than 0.05 was identified as significant.

## 3. Results

### 3.1. Clinical Assessment

Breast augmentation and related remodeling profiles using DIs and AFG were successfully performed in all patients (DIs and AFG). Follow-ups were performed in all patients (DIs and AFG) after baseline (T0) at 1 week (T1), 3 weeks (T2), 7 weeks (T3), 3 months (T4), 6 months (T5), 12 months (T6), and then annually until the third year after the last procedure. Several patients were not available to return to the control 2 years later. As a matter of fact, after the third year, 25 patients (45.4%) of the DI group and 28 patients (56%) of the AFG group were controlled at the fourth year, whereas 10 patients (18%) of the DI group and 12 patients (24%) of the AFG group were controlled at the fifth year. The mean follow-up was 36 months (range 12–60 months).

**Figure 5:**
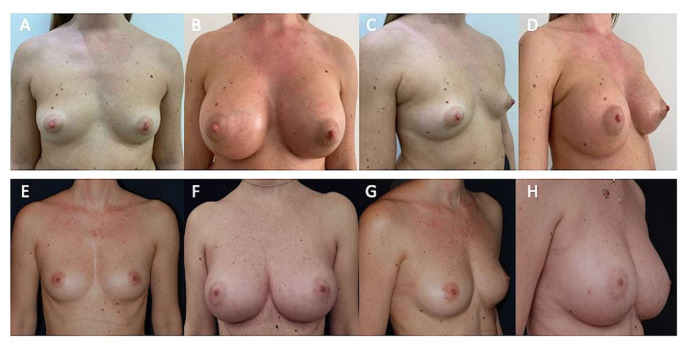
Patients shown in Figure 1 (definitive implants) and in Figure 2 (autologous fat grafting) in comparison. (**A**) Pre-operative projection in frontal view. The patient shown in Figure 1 affected by a moderate grade of bilateral breast hypoplasia. (**B**) Post-operative projection after 1 year (T6) in frontal view. Patient treated with 320 cc definitive implant, positioned in the sub-glandular plane, bilaterally. (**C**) Pre-operative projection in ¾ right view. (**D**) Post-operative projection after 1 year (T6) in ¾ left view. (**E**) Pre-operative projection in frontal view. The patient shown in Figure 2 affected by a moderate grade of bilateral breast hypoplasia. (**F**) Post-operative projection in frontal view after 1 year (T6) and two procedures based on ASC-enriched AFG. An increase of 72.8 mm in the three-dimensional volume after 1 year (T6) was observed, evidencing a cosmetic result comparable with that obtained by definitive implants (shown in Figure 5B). (**G**) Pre-operative projection in ¾ right view. (**H**) Post-operative projection in ¾ right view after 1 year (T6) and two procedures based on ASC-enriched AFG evidencing the naturalness of the outcomes.

A total of 89% (n = 49) of patients treated with DIs (Figure 1A–C and Figure 5A,C) showed excellent cosmetic results at T6 (after 1 year) (Figure 1D–F and Figure 5B,D), compared with the AFG patients, who showed the same results in only 64% (n = 32) of cases. The breast augmentation maintenance and contour restoration in the DI group was higher than that in the AFG group (*p* < 0.0001 vs. AFG group). More natural results in the AFG group was higher than that in the DI group (*p* < 0.0001 vs. DI group).

In 76% (n = 38) of breast augmentation patients treated with ASC-enriched AFG (Figure 2A,D and Figure 5E,G), we observed excellent cosmetic results and restoration of the breast contour and an increase of 43.3 mm in the three-dimensional volume after 3 weeks (T2), 29.5 mm after 6 months (T5) (Figure 2B,E and Figure 4B–D), and 25.7 mm after 12 months (T6), analyzed by MRI with 3-mm-thick slices and also by clinical comparison between pre- and post-op. A comparable result to that of DIs at 1 year (T6) was obtained in the AFG patients treated with two procedures based on ASC-enriched AFG. An increase of 72.8 mm in the three-dimensional volume after 1 year (T6) was observed (Figure 2C,F), evidencing a cosmetic result comparable with that obtained by the DIs (Figure 5F,H).

All people who underwent the treatments (DIs and AFG) reported being satisfied with the resulting texture, softness, and volume contours. In both groups (DI and AFG), most people were satisfied with the outcomes (*p* = 0.403), and they would undergo the procedure again (*p* > 0.787) and recommend the treatment to friends (*p* = 0.371) (Table 4). Regarding the self-evaluation of cosmetic results in terms of augmentation outcomes after 1 year, scores ranged from 3 to 6 in the AFG group and from 1 to 4 in the DI group (*p* = 0.033). The results displayed a trend in DI patients to be more satisfied than AFG patients (Table 4). Satisfaction grade assessment questionnaire analysis revealed that all patients in both groups (DI and AFG) would choose to undergo breast augmentation with fat injections or definitive implants, and that they were sufficiently informed about the risks and complications of these treatments (included the risk of fat graft resorption, the high possibility of repeating the treatment more times in the AFG group, and the risk of implant displacement and rejection in the DI group). When satisfaction grade was evaluated via a Visual Analog Scale (VAS), members of the DI and AFG groups were similarly satisfied (*p* = 0.21). Figure 5 shows patients that were categorized as showing “improvement” by all peers. When computing the new scores, patients in the DI group and the AFG group received respective scores (average) of 4.9 and 2.8 (*p* = 0.33) and, therefore, the DI group was regarded as presenting better improvement. Additionally, the absence of an inframammary horizontal fold scar (5 cm average) in the AFG group appeared to be common in the outcomes. As expected, patient satisfaction with the appearance of the scar was higher in the AFG group, in which the scars were 2 mm in maximum diameter and were hardly visible.

### 3.2. Breast and Chest Deformity Influence Aesthetic Outcomes

With more than 10 years in practice, the team independently evaluated the preop- and post-operative images (plastic surgeon), radiological findings (radiologist and plastic surgeon), and satisfaction assessments (plastic surgeon, radiologist, and psychologist). The intraclass correlation coefficient (ICC) was also calculated to assess the inter-observer reliability.

Breast and/or chest deformities were detected in 23 DI patients (42%) (9 patients with major complicating factors, 3 with a combination of two or more minor factors, and 11 with only one minor factor) and 27 AFG patients (54%) (9 patients with major complicating factor and 8 with a combination of two or more minor factors and 10 with only one minor factor).

Eleven patients of the DI group had suboptimal surgical results, compared with only three AFG patients. The deformities influenced more negatively the DI group outcomes than those of the AFG group (DCC = 0.081, *p* < 0.001).

The ICC was found to be good and statistically significant for inter-observer reliability (ICC = 0.083, *p* < 0.001), showing enough consistency among the three observers.

### 3.3. Operative Outcomes

The mean number of sessions required for ASC-enriched AFG was two. Only one session (mean transfer volume, 180 mL for breast, range 80–280 mL) was sufficient in 28 cases (56%). A second session (mean transfer volume, 130 mL for breast, range 80–180 mL) was necessary in the remaining 22 cases (44%). The formation of oil cysts was the only complication that occurred in the AFG group, detected in seven cases (14%) via US. A second look was not performed for side effects.

The mean number of interventions for DIs was one. Only one session (mean implant size volume, 235 mL for breast, range 150–320 mL) was sufficient in 48 cases (87%).

Fifty-two patients were treated with smooth DIs, while three patients received anatomic implants. A second look was performed for side effects in seven cases (13%) (mastopexy for breast ptosis, n = 1; implant replaced for inadequate final volume, n = 2; surgery for capsular contracture, n = 4).

### 3.4. Study Limitations

Performing a deep analysis of the results obtained during this investigation, several limitations led to the presence of bias in the present work. Firstly, regarding the AFG group, the lack of a single standardized protocol for the isolation methods of ASCs was highlighted, as well as the lack of standardized enrichment procedures. Two enrichment procedures of AFG based on mechanical ASC isolation (centrifugation, filtration) and on the digestion method (enzymatic) were analyzed. This difference may influence the fat maintenance percentage. Secondly, regarding the DI group, the differences in terms of implant positioning (sub-muscular or sub-glandular), incision (peri-areolar or inframammary), and features of the implants (round or anatomic) made the group non-homogeneous.

In each case, the bias was limited by the “custom made approach” for every patient, in which the choice of the definitive prosthesis and the related positioning plane was made based on the tissues available (sufficient gland = sub-glandular plane; very thin thickness grade of the gland and related tissues = sub-muscular plane; low thickness grade of glands and soft tissues = dual plane).

The follow-up was completed in all patients (DIs and AFG) until the third year after the last procedure. Several patients were not available to come back to the control the two years later, showing a low follow-up rate after five years (45.4% of the DI group and 56% of the AFG group were controlled at the fourth year, whereas only 18% of the DI group and 24% of the AFG group were controlled at the fifth year).

The present investigation is the first retrospective case-control study in this field, and for this reason, additional perspectives studies and/or controlled trials will be necessary.

## 4. Discussion

The presence of breast or chest wall deformities will make the surgical procedure more challenging and affect the cosmetic outcome. For this reason, these deformities should be accurately considered during pre-operative evaluation. Breast asymmetry is the most common deformity found. Its incidence was reported to be 88% by Rohrich et al. [10] (n = 100) and 81.1% by DeLuca-Pytell et al. [11] (n = 375).

The analysis of major and minor complicating factors has generally been done according to the implication degree and related effect produced by a specific deformity on the aesthetic outcomes. In the present study, major complicating factors were defined as deformities that could, if presented alone, lead to a suboptimal result, while the minor factors were those that led to a suboptimal outcome only if they were combined. A single minor complicating factor has less of an effect on the outcome and might even pass unnoticed if not corrected. In this retrospective study, every major complicating factor was directly correlated to a suboptimal result. Additionally, only the combination of two or more minor complicating factors led to a suboptimal result. It is necessary to highlight that the suboptimal results in the present study were not defined based on patient satisfaction because there were patients satisfied with suboptimal results, with a significant correspondence between 11 DIs patients and three AFG patients that presented a “good” satisfaction level. The complicating factors are presented in Table 2, yet it is possible that additional major or minor complicating factors such as kyphosis or sunken chest can exist in other surgeons’ series. The relationship between BMI and breast augmentation has not been studied enough in the literature. Some works have shown that patients with a high or low BMI have more post-operative side effects [12,13]. In the current study, patients with a low BMI presented a diminished skin envelope, making the treatment challenging both for DIs and for AFG. Pectus excavatum is another deformity that can result in worse aesthetic outcomes, especially when DIs are used. The right option for camouflaging this deformity is the AFG. Scoliosis deformity, even if subtle, may present as breast asymmetry [14]. There is a correlation between the severity of scoliosis and the difference in breast volumes [15]. The NAC position may be asymmetric on both the horizontal and vertical planes. An NAC asymmetrically placed on the horizontal axis was first reported by Khan [16], who described a related incidence of 12%. Breast augmentation may result in more prominent nipple lateralization.

Regarding the breast volume evaluation, it appears fundamental during the pre-operative phase to identify the anatomic regions needing correction, performing breast volume analysis, shape, and symmetry evaluation. Kayar et al. [17] compared five methods for breast volume estimation: mammographic, anthropometric, the Grossman–Roudner device, the Archimedes procedure, and the casting technique, demonstrating that mammographic evaluation seems to be much more accurate. Moreover, three-dimensional (3D) evaluation with MRI proved to be an alternative method that is accurate and effective for volume estimation by depicting in vivo breast shape and symmetry. Using MRI any deformity, asymmetry, and post-operative changes can be correctly located, estimated, and evaluated as well as loss of fat volume. Breast volume modification and shape changes may be compared during the follow-up thanks to the reproducibility of the assessment [17,18].

Regarding the outcomes obtained in terms of breast volume maintenance, 89% of patients treated with DIs showed excellent cosmetic results at 1 year, compared with the patients of the AFG group, who showed the same results in only 64% of cases. The breast augmentation maintenance and contour restoration in the DI group were higher than in the AFG group (*p* < 0.0001 vs. AFG group). By contrast, more natural results in the AFG group were higher than in the DI group (*p* < 0.0001 vs. DI group). When the satisfaction grade was evaluated through a VAS, members of the DI and AFG groups were similarly satisfied (*p* = 0.23). The outcomes appeared to be similar and in line with those shown by Brault et al. [19], in the only article published previously (2017) on the comparison of DI vs. AFG in breast augmentation. In detail, they stated “*Surprisingly, there was no significant difference between the implant group and the lipofilling group concerning the ‘feel of the breast*.’ *In both groups, the highest scores were for physical wellbeing*.”

In the present retrospective study, a high level of satisfaction in the DI and AFG groups was also evaluated using the VAS scale, where in the DI group the long-term breast volume maintenance and the possibility of having a definitive result with only one procedure were the most important advantages, compared with the AFG group, in which the more natural results and the lack of second-look treatment for side effects were observed. Additionally, the absence of an inframammary fold scar in the AFG group appeared common in the outcomes. As expected, patient satisfaction with the appearance of the scar was higher in the AFG group.

Despite the more evident and lasting result obtained by the DIs and despite the appeal of the AFG technique based on a more natural result, some problems remained concerning both procedures.

In the case of DIs, a silicone-filled biomaterial is still considered a foreign body, even though it has physiological inertness, low toxicity, and anti-adhesive properties, while the possible related side effects, such as rejection and/or displacement and/or capsular contracture, appeared to be the major concerns.

In the case of AFG, although it is an autologous bioactive material scaffold without the major concerns observed in DIs, the final breast volume maintenance, the application of a standardized injection technique, and the necessity of repeating the treatment in some cases appeared to be the major limits.

Currently, only one study on the tuberous breast (and not in breast hypoplasia), published by Brault et al. [19], compared the results obtained by DIs with AFG in breast treatment.

The retrospective design of this study is an important limitation. A prospective analysis would lead to a higher level of evidence, as benefits could be measured by comparing pre-operative and post-operative differences between each group by using the pre- and post-operative Breast-Q scale. Since it is specifically for the DI group and not for the AFG group (several questions were not appropriate for AFG), this latter procedure was inapplicable. On the other hand, the three-year follow-up here analyzed is the most prolonged in the literature of the present investigation so far.

## 5. Conclusions

Complicating factors in breast augmentation must be identified during the pre-operative evaluation, aiming to recognize challenging cases and to plan a more adequate surgical procedure. Breast deformities (volume asymmetry and tuberous breast, high grade of hypoplasia, unilateral breast hypoplasia, NAC asymmetry), chest wall deformities (pectus excavatum and carinatum), and low BMI may compromise the cosmetic outcome, being major complicating factors. AFG treatment determined more natural results, allowing also better results compared with DIs, patients with pectus excavatum and/or carinatum, volume asymmetry, and unilateral breast hypoplasia. DI treatment determined a larger breast volume after a single procedure (compared with the two requirements for AFG to obtain the same DI volume) and a lasting result in almost all patients. A prospective study appears necessary in the immediate future, aiming to better evaluate and understand the post-operative results related to patient satisfaction.

## Figures and Tables

**Table 1:** Patients’ data and clinical assessment.

	DI Group	AFG Group
	(DIs)	(AFG)
**Number of patients, no°**	55	50
**Age at surgery, yr.**	39.5 (min 18, max 61)	38.0 (min 18, max 58)
**BMI at surgery, kg/m^2^**	26.5 (min 18, max 35)	26.5 (min 18, max 35)
**Bilateral Hypoplasia**	30 (moderate),	28 (moderate),
	10 (high),	7 (high),
	12 (low)	10 (low)
**Unilateral Hypoplasia**	3	5
**Pre-menopausal**	40 (72%)	38 (76%)
**Only one session**	48 (87%)	28 (56%)
**Mean transfer volume for each breast**	235 mL (Range 150–320 mL)	180 mL (Range 80–280 mL)
**Second session**	7 (13%)	22 (44%)
	mastopexy n = 1;	130 mL
	implant replaced n = 2;	(Range 80 mL–180 mL)
	surgery for capsular	
	contracture n = 4	
**Volume maintenance percentage (1 year later)**	100% (All patients)	70.8% ± 5% (All patients)
**Volume maintenance percentage (3 years later)**	100% (48 patients)	58% ± 8% (All patients)
**Cyst formation and Calcification**	0	7 (14%)
**Fat Necrosis**	0	0
**Breast ptosis**	1 (1.8%)	0
**Inadequate final volume**	2 (3.6%)	0
**Capsular contracture**	4 (7.2%)	0

**Table 2:** Breast and chest deformities.

Deformity	Grade	DI Patients (55)	AFG Patients (50)
**Bilateral breast** **Hypoplasia (BBH)**	High	10	7
	Moderate	30	28
	Low	12	10
**Unilateral breast** **Hypoplasia (UBH)**	Moderate	3	5
**Vertical nipple** **Asymmetry**	Low	2 (included in BBH patient moderate grade)	4 (included in BBH patient moderate grade)
**Tuberous breast**	Moderate	2 (included in UBH patient)	2 (included in UBH patient) 6 (included in BBH patient moderate grade)
**Pseudoptosis**	Low	3 (included in BBH patient low grade)	3 (included in BBH patient low grade)
**Pectus excavatum**	Moderate	2 (included in BBH patient moderate grade)	4 (included in BBH patient moderate grade)
	Low	2 (included in BBH patient moderate grade	2 (included in BBH patient high grade)
**Pectus carinatum**	Low	0	3 (included in BBH patient moderate grade)

**Table 3:** Complicating factors classification.

Deformity	DI Group	AFG Group
**Major complicating Factors**	NAC asymmetry	NAC asymmetry
	Volume asymmetry	-
	Unilateral breast hypoplasia	-
		High grade of breast hypoplasia
	Pectus excavatum	-
	Pectus carinatum	-
	-	Low BMI
**Minor complicating** **Factors**	-	Pseudoptosis
	Unilateral Tuberous breast	-
	Scoliosis	-

**Table 4:** Patient satisfaction data.

	DI Group	AFG Group
**Patients no°**	55	50
Self-evaluation of cosmetic results	**49 (Fully Satisfied)**:	**38 (Fully Satisfied)**:
(Score range 1–6/excellent-very poor)	29 (Excellent/1)	25 (Excellent/1)
	15 (Very good/2)	10 (Very good/2)
	11 (Good/3)	3 (Good/3)
	2 (Not/Fully/Satisfied):	9 (Not/Fully/Satisfied):
	2 (Sufficient/4)	9 (Sufficient/4)
	4 (Not/Satisfied):	3 (Not/Satisfied):
	3 (Poor/5)	3 (Poor/5)
	1 (Very poor/6)	0 (Very poor/6)
Satisfaction with the result	51	47
Recommend the treatment to a friend	50	45
Available to breast augmentation	55	50
Sufficiently informed about risks and complications	55	50

## Appendix A. Patient’s Satisfaction Questionnaire

Degree of Satisfaction:

1 - Excellent;

2 - Very good;

3 - Good;

4 - Sufficient;

5 - Poor;

6 - Very poor.

In the pre-operative period

According to the scale above, grade questions 1 through 6 according to your degree of satisfaction:

1. How do you feel about your breast shape?

2. How do you feel about your breast volume?

3. How do you feel about your breast symmetry?

4. How do you feel about your breast softness?

5. How do you feel about your breast texture?

6. Did you have any informations on risk and complication (included the risk of reabsorption of fat graft and the high possibility to repeat the treatment more times)?

In the post-operative period at 1 week (T1), 3 weeks (T2), 7 weeks (T3), 3 months (T4), 6 months (T5), 12months (T6) and then annually.

According to the scale above, grade questions 1 through 6 according to your degree of satisfaction:

1. How do you feel about your breast shape?

2. How do you feel about your breast volume?

3. How do you feel about your breast symmetry?

4. How do you feel about your breast softness?

5. How do you feel about your breast texture?

6. Did you have any informations on risk and complication (included the risk of reabsorption of fat graft and the high possibility to repeat the treatment more times)?
